# Association of soluble TREM2 with Alzheimer’s disease and mild cognitive impairment: a systematic review and meta-analysis

**DOI:** 10.3389/fnagi.2024.1407980

**Published:** 2024-05-22

**Authors:** Ruiqi Wang, Yijun Zhan, Wenyan Zhu, Qianwen Yang, Jian Pei

**Affiliations:** Department of Acupuncture, Longhua Hospital, Shanghai University of Traditional Chinese Medicine, Shanghai, China

**Keywords:** soluble TREM2, Alzheimer’s disease, mild cognitive impairment, neuroinflammation, meta-analysis

## Abstract

**Objective:**

Soluble triggering receptor expressed on myeloid cells 2 (sTREM2) is a potential neuroinflammatory biomarker linked to the pathogenesis of Alzheimer’s disease (AD) and mild cognitive impairment (MCI). Previous studies have produced inconsistent results regarding sTREM2 levels in various clinical stages of AD. This study aims to establish the correlation between sTREM2 levels and AD progression through a meta-analysis of sTREM2 levels in cerebrospinal fluid (CSF) and blood.

**Methods:**

Comprehensive searches were conducted in PubMed, Embase, Web of Science, and the Cochrane Library to identify observational studies reporting CSF and blood sTREM2 levels in AD patients, MCI patients, and healthy controls. A random effects meta-analysis was used to calculate the standardized mean difference (SMD) and 95% confidence intervals (CIs).

**Results:**

Thirty-six observational studies involving 3,016 AD patients, 3,533 MCI patients, and 4,510 healthy controls were included. CSF sTREM2 levels were significantly higher in both the AD [SMD = 0.28, 95% CI (0.15, 0.41)] and MCI groups [SMD = 0.30, 95% CI (0.13, 0.47)] compared to the healthy control group. However, no significant differences in expression were detected between the AD and MCI groups [SMD = 0.09, 95% CI (−0.09, 0.26)]. Furthermore, increased plasma sTREM2 levels were associated with a higher risk of AD [SMD = 0.42, 95% CI (0.01, 0.83)].

**Conclusion:**

CSF sTREM2 levels are positively associated with an increased risk of AD and MCI. Plasma sTREM2 levels were notably higher in the AD group than in the control group and may serve as a promising biomarker for diagnosing AD. However, sTREM2 levels are not effective for distinguishing between different disease stages of AD. Further investigations are needed to explore the longitudinal changes in sTREM2 levels, particularly plasma sTREM2 levels, during AD progression.

**Systematic review registration:**

https://www.crd.york.ac.uk/prospero/display_record.php?ID=CRD42024514593

## 1 Introduction

Triggering receptor expressed on myeloid cells 2 (TREM2), a receptor on microglial membranes, has emerged as a research focus in Alzheimer’s disease (AD), supported by the positive results of genome-wide association studies (GWAS) over the past decade (Bertram and Tanzi, 2019). Microglia dysfunction in the brain due to TREM2 risk variants, along with neuroinflammation can increase the risk of AD (Wang et al., 2024). Specifically, the rs75932628 (p.R47H) dysfunction variant of TREM2 has been identified as a major genetic risk factor, showing a significant association with AD in a meta-analysis of over 168,000 Greek populations (Korvatska et al., 2015; Rikos et al., 2022). Soluble TREM2 (sTREM2), the cleaved extracellular portion of TREM2 by metalloproteinases, is detectable in both cerebrospinal fluid (CSF) and blood (Deming et al., 2019). The incidence of all-cause dementia is notably increased with higher sTREM2 levels, including AD, vascular dementia (VaD) (Ohara et al., 2019), frontotemporal dementia (FTD) (Schulz et al., 2021) and dementia with Lewy bodies (DLB) (Morenas-Rodríguez et al., 2019). Its levels, particularly in CSF, possess significant diagnostic potential for differentiating patients with cognitive impairment from healthy individuals and serve as reliable indicators of cognitive decline and neuroinflammation in neurodegenerative diseases (Španić et al., 2023).

Neuroinflammation is recognized as a pathological hallmark of mild cognitive impairment (MCI) and AD. However, it alone is not a specific pathological marker, and its biomarkers, including TREM2, face limitations due to a lack of conclusive evidence (Kandiah et al., 2022). Previous research has underscored the value of CSF and blood sTREM2 levels as biomarkers for predicting disease progression in AD, although results have been inconsistent. A meta-analysis revealed elevated CSF sTREM2 levels in both AD and MCI patients, while no association was found between plasma sTREM2 levels and the risk of AD development (Gu et al., 2023). Recent studies focusing on sTREM2 in the progression of AD have advanced rapidly. Considering the better clinical operability of blood biomarkers, recent research has increasingly focused on the expression of sTREM2 in plasma and has identified significantly elevated levels in AD patients (Schulz et al., 2021; Zhao et al., 2022; La Rosa et al., 2023).

This meta-analysis aims to reassess the CSF and blood levels of sTREM2 in patients with MCI and AD and to explore the impact of sTREM2 on AD progression. It combines up-to-date data from relevant observational studies to test the hypothesis that elevated sTREM2 levels are associated with cognitive decline. These findings could provide insights into the underlying biomechanisms of AD and provide new diagnostic and therapeutic approaches.

## 2 Methods

The protocol is registered at the International Prospective Register of Systematic Reviews (PROSPERO) (registration number: CRD42024514593).

### 2.1 Search strategy

Two investigators (RW and WZ) conducted independent searches in the PubMed, Embase, Web of Science, and Cochrane Library databases for articles published up to 15 February 2024. The main keywords included (soluble TREM2 OR sTREM2) AND (Alzheimer’s Disease OR Senile Dementia OR AD OR Mild Cognitive Impairment OR MCI), plus additional relevant keywords as outlined in Supplementary Table S1. Additionally, the reference lists of prior studies were extensively examined.

### 2.2 Selection criteria

The inclusion criteria were: (1) studies assessing CSF or blood (plasma or serum) sTREM2 in AD patients, MCI patients, or healthy controls. (2) Utilization of international diagnostic criteria for AD and MCI were reported. (3) Availability of sTREM2 data for both disease and control groups. The exclusion criteria included (1) studies lacking precise sTREM2 levels, even after contact with the corresponding author; and (2) reviews, abstracts, case reports, letters, and commentaries.

### 2.3 Data extraction

Following predefined criteria, two reviewers (QY and WZ) independently extracted data from the selected studies, including the first author’s name, publication year, country, sample size, diagnostic criteria, mean age, and gender distribution, CSF and blood sTREM2 levels, and the assay method. For studies providing range data, means and standard deviation (SD) were calculated (Hozo et al., 2005). The data from each eligible study were compiled into a spreadsheet.

### 2.4 Quality assessment

The quality of the observational studies was assessed using the Newcastle-Ottawa scale (NOS), which comprises 3 sections totaling 8 entries with a maximum score of 9; a score of ≥7 was deemed high quality (Stang, 2010). Two reviewers (YZ and QY) performed the quality assessments independently; in cases of disagreement, a third reviewer (JP) was consulted to resolve the issue.

### 2.5 Statistical analysis

All the statistical analysis were performed using STATA version 14.0 (StataCorp LLC, College Station, TX, United States). The sTREM2 levels in the cognitively impaired patients and controls were assessed by calculating the combined standardized mean difference (SMD) and 95% confidence interval (CI). Interstudy heterogeneity was calculated using Higgins’s I-squared test based on Cochrane’s Q. A random-effects model was employed if I^2^ ≥ 50%, indicating statistical heterogeneity; otherwise, a fixed-effects model was used. Additionally, subgroup analysis and meta-regression of the mean age, assay method, diagnostic criteria, and proportion of women were conducted to explore sources of heterogeneity. Sensitivity analysis was performed by systematically excluding each study to ensure the reliability of the combined estimates. Moreover, publication bias was assessed using Egger’s test with the trim and fill method. A *p* < 0.05 was considered to statistically significant.

## 3 Results

### 3.1 Literature selection

**Figure 1:**
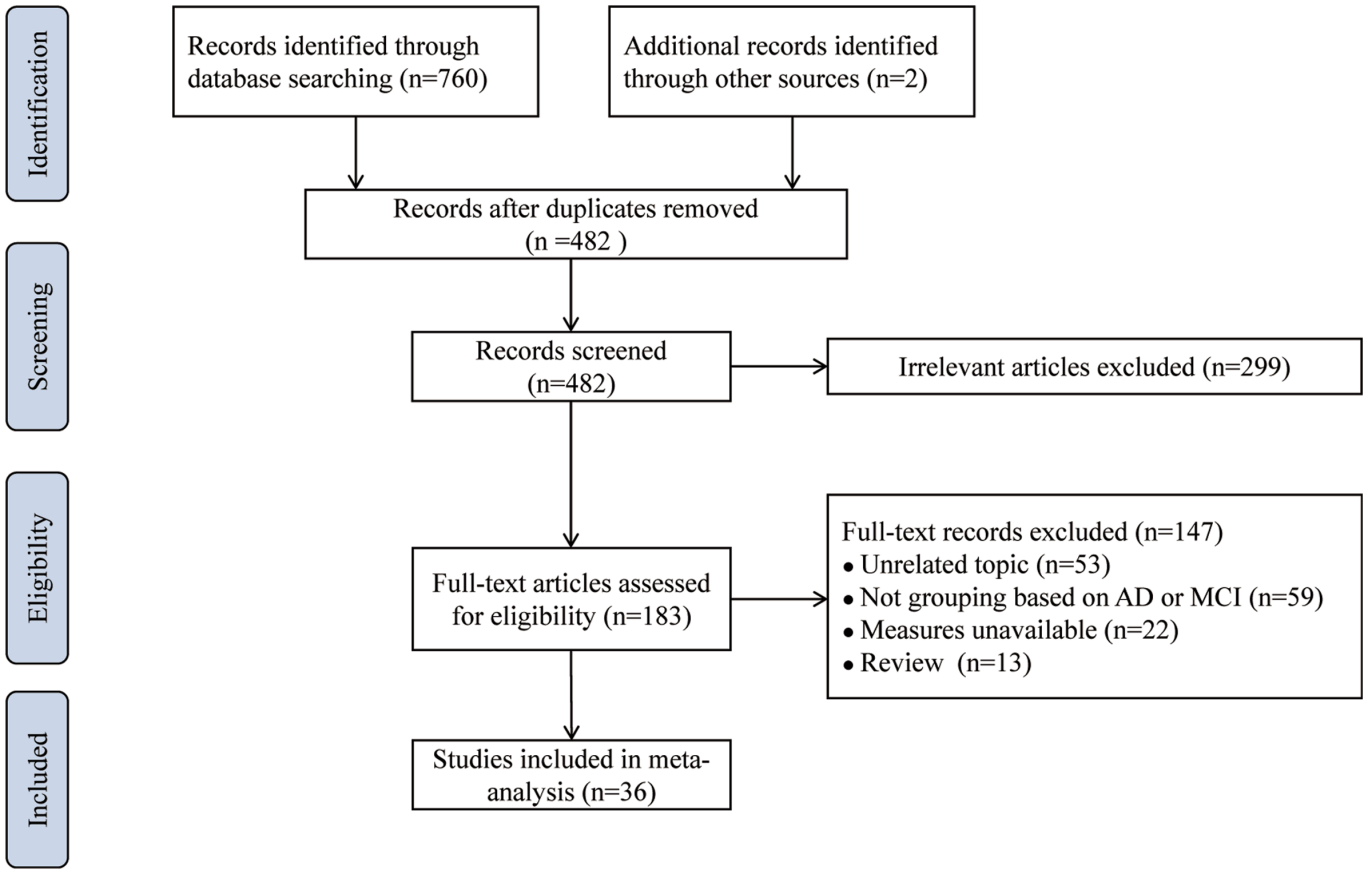
Flow chart of the study selection process and results.

Initially, 762 potentially relevant studies were retrieved. After removing 280 duplicates, 299 articles were extracted by screening titles and abstracts, and 147 studies were excluded after full-text review. Ultimately, thirty-six articles were included. The overall screening process and results are presented in Figure 1.

### 3.2 Study characteristics

The thirty-six articles (Hu et al., 2014, 2021a; Kleinberger et al., 2014; Gispert et al., 2016, 2017; Henjum et al., 2016; Heslegrave et al., 2016; Piccio et al., 2016; Suárez-Calvet et al., 2016; Bekris et al., 2018; Brosseron et al., 2018; Deming et al., 2019; Ewers et al., 2019; Morenas-Rodríguez et al., 2019; Nordengen et al., 2019; Banerjee et al., 2020; Edwin et al., 2020; Ferri et al., 2020; Franzmeier et al., 2020; Knapskog et al., 2020; Diaz-Lucena et al., 2021; Ma et al., 2021; Schulz et al., 2021; Van Hulle et al., 2021; Chen et al., 2022; Li et al., 2022; Shi et al., 2022; Winfree et al., 2022; Zhao et al., 2022; Finze et al., 2023; Giannisis et al., 2023; Hok-A-Hin et al., 2023; La Rosa et al., 2023; Paolini et al., 2023; Španić et al., 2023; Wang et al., 2023) were all published between 2014 and 2023, showed an increase in publications from 2017 to 2023. These studies involved 3,016 AD patients, 3,533 MCI patients, and 4,510 healthy controls, primarily conducted in Europe and the United States. Thirty studies (encompassing 32 comparisons) examined the relationship between CSF sTREM2 and AD, while 20 focused on the association with MCI. Additionally, eight studies explored plasma sTREM2 expression in AD patients. Five studies detected both CSF and plasma sTREM2 levels. Seventeen studies measured CSF sTREM2 levels in both AD and MCI patients, but only two assessed plasma sTREM2 levels. Twenty-four studies employed enzyme-linked immunosorbent assay (ELISA) as the primary assay method. The NOS score ranged from 6 to 8, with most studies classified as high quality and three as moderate quality (Supplementary Table S2).

### 3.3 Association between sTREM2 and AD progression

**Figure 2:**
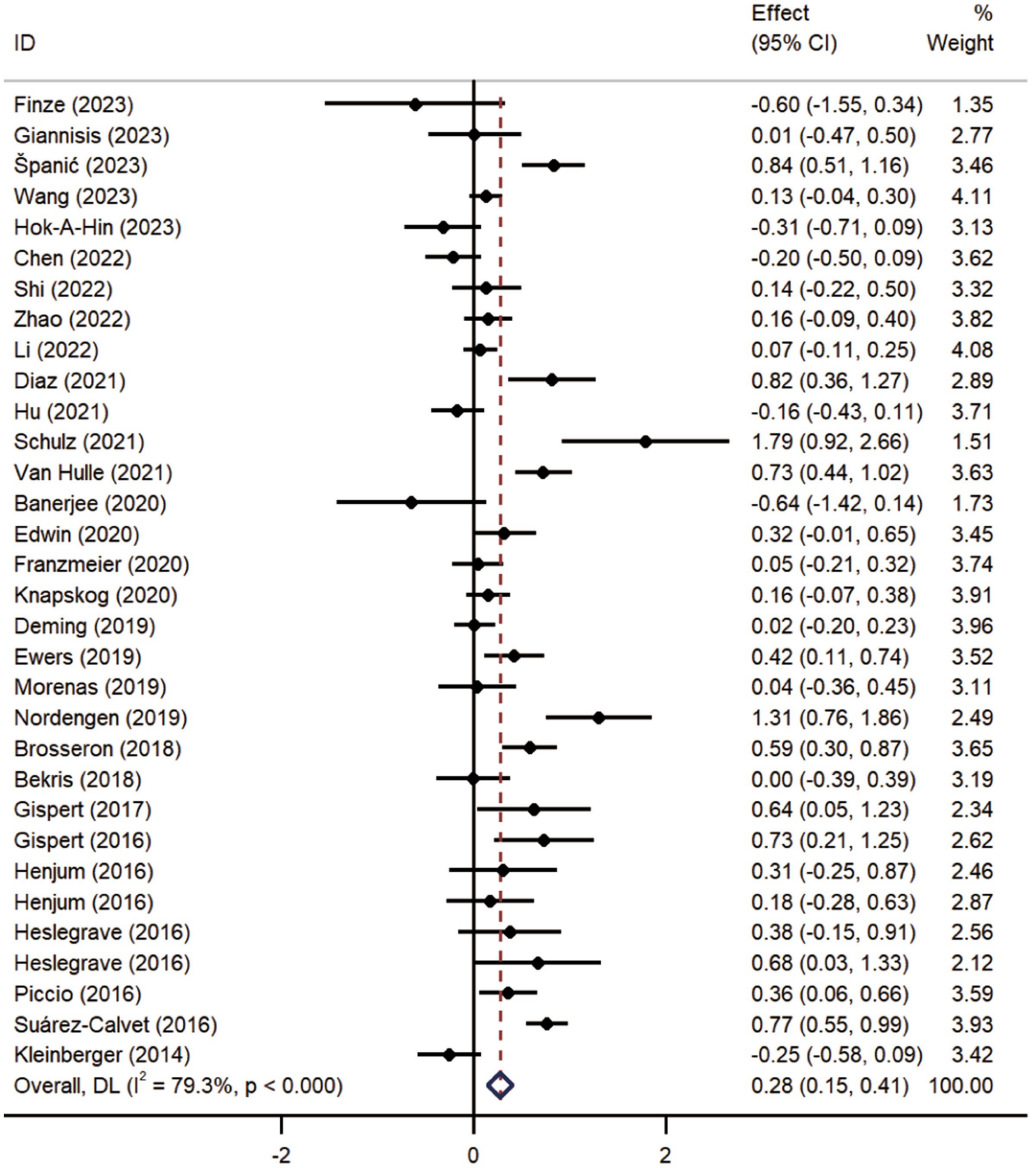
Forest plot of CSF sTREM2 levels in patients with AD compared to controls. Weights are from random-effects model.

**Figure 3:**
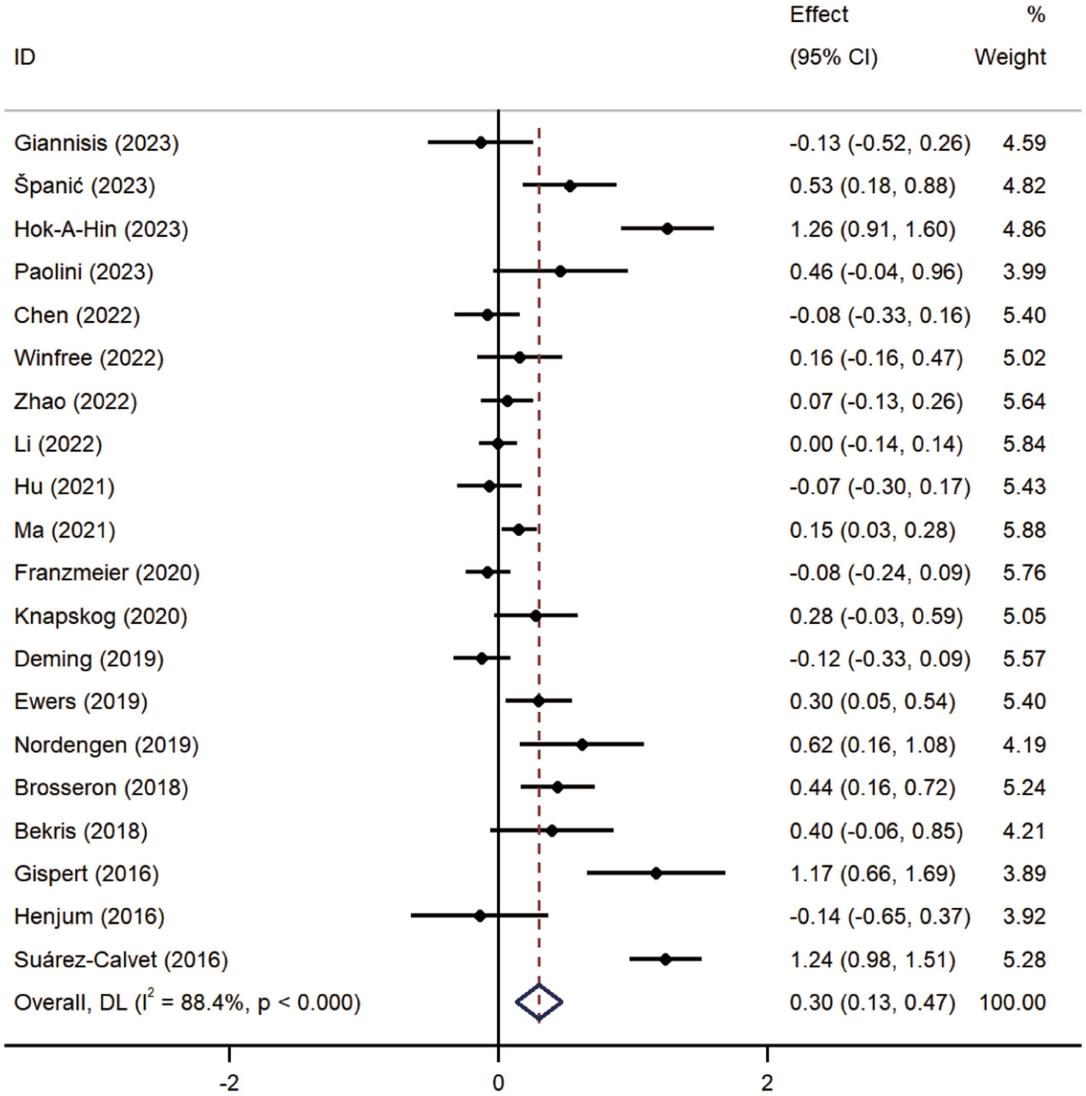
Forest plot of CSF sTREM2 levels in patients with MCI compared to controls. Weights are from random-effects model.

**Figure 4:**
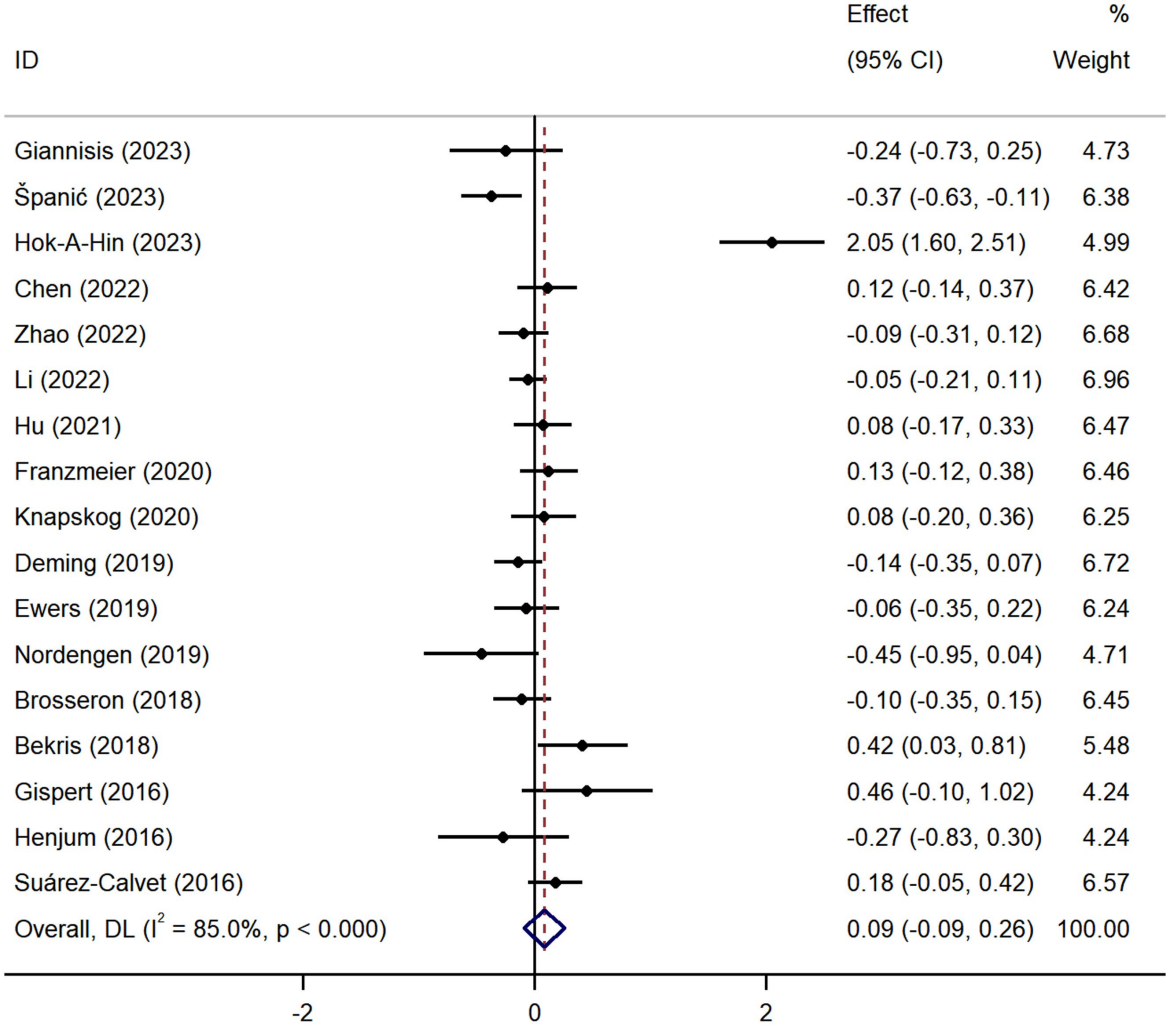
Forest plot of CSF sTREM2 levels in patients with AD compared to MCI patients. Weights are from random-effects model.

**Figure 5:**
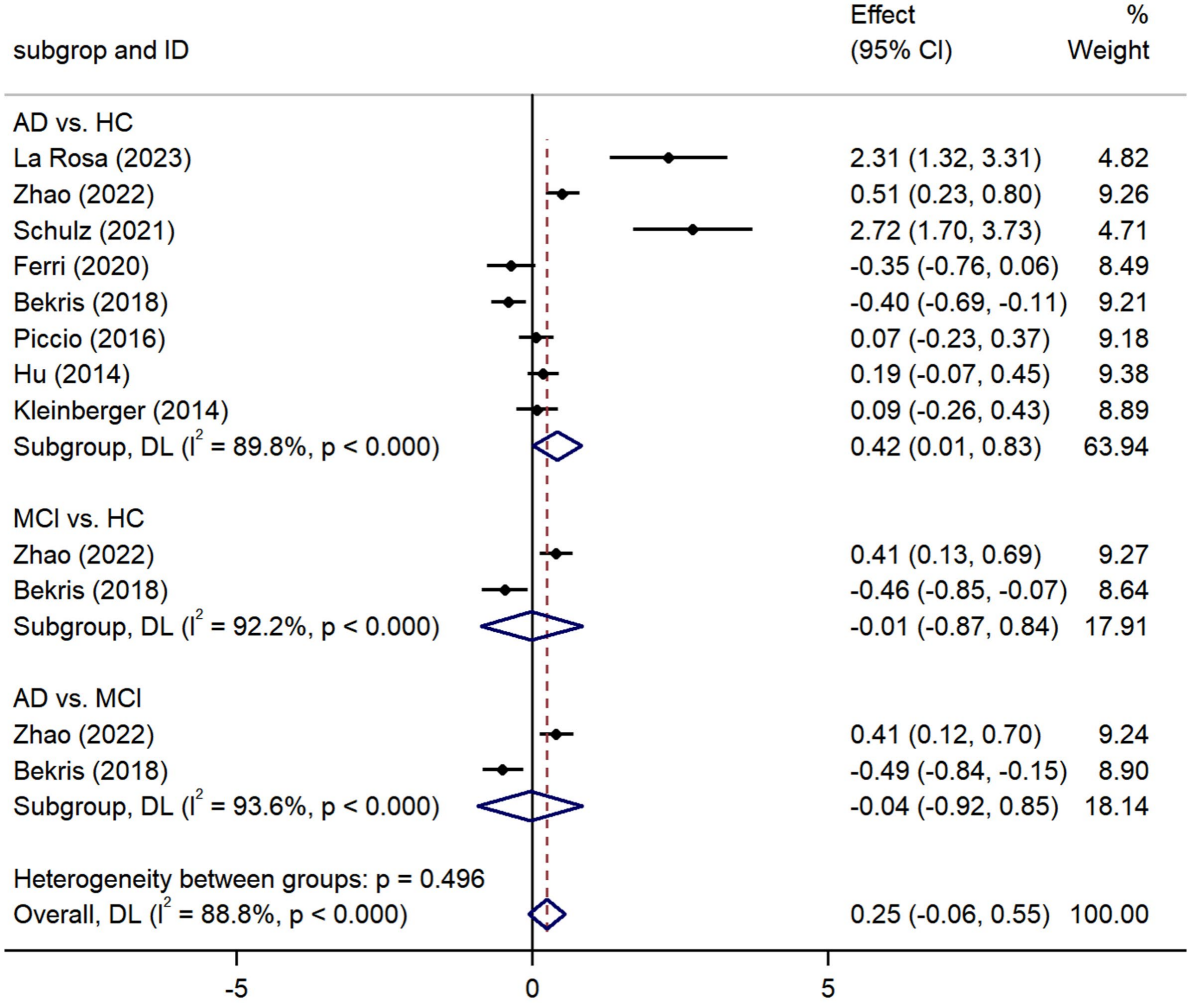
Forest plot of plasma sTREM2 expression levels in patients with AD and MCI. Weights and between-subgroup heterogeneity text are from random-effects model.

Pooled analysis showed significantly higher CSF sTREM2 concentrations in patients in both the AD [SMD = 0.28, 95% CI (0.15, 0.41)] and MCI [SMD = 0.30, 95% CI (0.13, 0.47)] groups compared to the HC group (Figures 2, 3). However, no significant difference in sTREM2 levels was observed between the AD and MCI groups [SMD = 0.09, 95% CI (−0.09, 0.26)] (Figure 4). For plasma sTREM2, elevated levels were found only in the AD group [SMD = 0.42, 95% CI (0.01, 0.83)] compared to the HC group (Figure 5). Due to high heterogeneity, random effects models were applied for the analysis.

### 3.4 Subgroup analysis and meta-regression analysis

**Figure 6:**
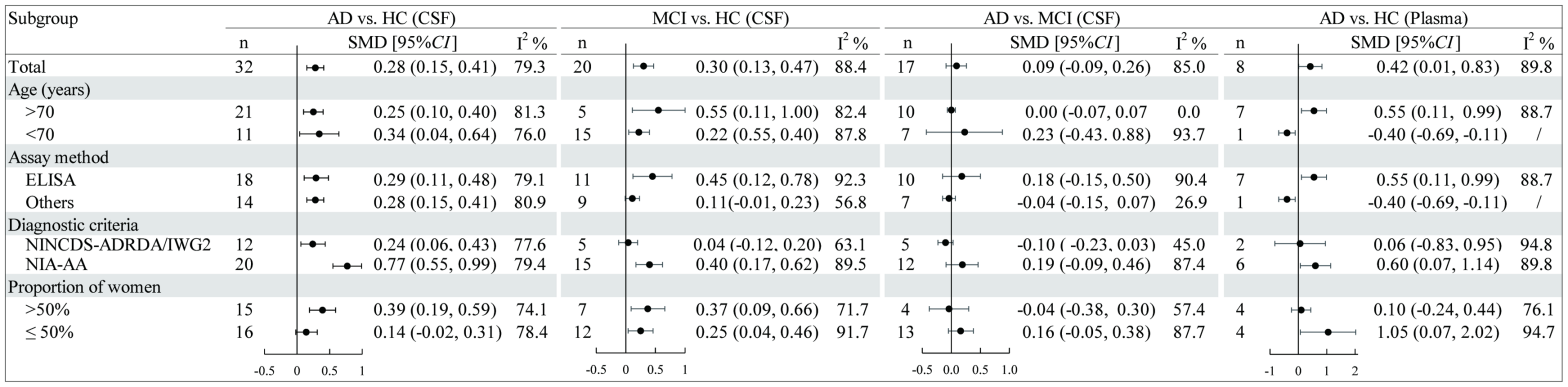
Subgroup analysis of CSF and plasma sTREM2 levels stratified by study characteristics.

As Figure 6 illustrates, the results of the subgroup analysis show elevated sTREM2 levels in the cognitive impairment group across different ages compared to the control group. ELISA tests confirmed that sTREM2 was elevated in the AD group regardless of the assay used, and also in the MCI group [SMD = 0.45, 95% CI (0.12, 0.78)]. Moreover, sTREM2 levels were higher in AD patients diagnosed using the NIA-AA criteria [SMD = 0.77, 95% CI (0.55, 0.99)] and in studies with a higher proportion of female participants [SMD = 0.39, 95% CI (0.19, 0.59)]. MCI patients also showed increased sTREM2 levels in studies using NIA-AA criteria [SMD = 0.40, 95% CI (0.17, 0.62)], with no significant differences in sTREM2 levels related to gender variation. According to the subgroup analysis of plasma sTREM2, higher sTREM2 levels were detected in AD patients aged over 70 years [SMD = 0.55, 95% CI (0.11, 0.99)], as assessed by ELISA [SMD = 0.55, 95% CI (0.11, 0.99)], diagnosed using the NIA-AA criteria [SMD = 0.60, 95% CI (0.07, 1.14)], and in studies with a lower proportion of female participants [SMD = 1.05, 95% CI (0.07, 2.02)]. None of the factors significantly reduced heterogeneity. Meta-regression results indicated that these factors did not fully explain the source of heterogeneity in other component comparisons (Supplementary Table S3).

### 3.5 Sensitivity analysis and publication bias

After excluding any of the referenced studies, sensitivity analysis showed no statistically significant change in the meta-analysis results, underscoring the findings’ relative reliability (Supplementary Figure S1). Publication bias was evaluated in each group’s meta-analyses. Egger’s test identified notable publication bias in the MCI vs. HC groups (*p* = 0.042), while no significant publication bias risk was found in the other groups (Supplementary Table S4). The trim and fill method indicated that no additional studies needed to be included, and the results remained unchanged [SMD = 1.35, 95% CI (1.14, 1.60)], demonstrating both stability and minimal impact from publication bias (Supplementary Figure S2).

## 4 Discussion

### 4.1 Summary of findings

A sustained and excessive inflammatory response is a common pathological basis for AD progression. Identifying relevant sensitive biomarkers is crucial for early AD diagnosis and intervention. Changes in sTREM2 concentrations in the CSF and blood may indicate neuroinflammation and microglial activation during AD development. This study found higher CSF sTREM2 levels in both MCI and AD groups but no differences between them. However, contrary to a previous meta-analysis (Gu et al., 2023), elevated plasma sTREM2 levels were also observed in AD patients. While no link was found between plasma sTREM2 levels and MCI risk, a recent study indicated that elevated plasma sTREM2 levels in MCI patients could increase the likelihood of developing AD (Zhao et al., 2022). These varying results may reflect different microglial responses to the pathological features at various AD stages.

Subgroup analysis revealed that AD patients over 70 years exhibited increased plasma sTREM2 levels, whereas CSF sTREM2 levels showed no age-related differences. This inconsistency may stem from variations in study inclusion criteria and small sample sizes, contradicting previous research that found a direct correlation between CSF sTREM2 levels and age in AD patients (Knapskog et al., 2020). A positive correlation exists between age and CSF sTREM2 level disparities in AD patients and HCs (Hu et al., 2021b). In most studies, there was a notable age discrepancy between the cognitively impaired group and the healthy controls, with half of the healthy control group having an average age of less than 65 years. In two other studies (Heslegrave et al., 2016; Banerjee et al., 2020), the average age of AD patients was less than 65 years. Previous studies have confirmed that patients with late-onset AD (LOAD) have higher peripheral blood TREM2 mRNA levels than those with early-onset AD (EOAD), suggesting that this differential expression is linked to age rather than AD disease status (Guven et al., 2020). Additionally, higher CSF sTREM2 levels were observed in studies with a high proportion of females. Gender-specific responses to TREM2 in inducing disease-associated microglial states were demonstrated in earlier research (Krasemann et al., 2017). A research team found that TREM2 expression levels were higher in female aged mice compared to male mice during pathway enrichment analysis of gender-specific gene expression (Ocañas et al., 2023). Genetic polymorphisms, such as the R47H mutation, may also influence gender differences, increasing AD risks predominantly in female mice (Sayed et al., 2021). However, the impact of gender on sTREM2 in clinical studies remains unclear (Piccio et al., 2016; Knapskog et al., 2020). ELISA is commonly used for sTREM2 detection, and the use of different kits may lead to heterogeneity. Moreover, sTREM2 values varied considerably across studies due to differences in analytical parameters and sample processing methods. The differences in findings among various studies may stem from differences in participant characteristics, including disease stage, all of which may introduce selection bias in the overall analysis.

### 4.2 Potential mechanism of sTREM2 in AD

TREM2 is an immunoglobulin superfamily transmembrane receptor mainly expressed by microglial cells in the brain (Piccio et al., 2016). Although it is generally thought that TREM2 acts protectively in AD, recent findings suggest that microglia with high TREM2 expression may be harmful (Rachmian et al., 2024). TREM2 activation may worsen Aβ-induced tau pathology, possibly accelerating its progression (Jain et al., 2023). Genetic variations significantly affect TREM2’s structure and function, and a GWAS has identified 46 TREM2 variants linked with AD that increase the risk of late-onset AD (Carmona et al., 2018). Without TREM2 mutations, TREM2 levels correlate with disease pathology accumulation (Perez et al., 2017). TREM2 levels are markedly increased in the brains of AD patients and transgenic mouse models (Jiang et al., 2014; Lue et al., 2015). It has been confirmed that TREM2 expression also rises with age in neuropathologically normal human brains (Forabosco et al., 2013). The extracellular domain of TREM2 is cleaved by disintegrin and metalloproteinase 10 (ADAM10) and ADAM17, releasing a soluble N-terminal extracellular domain known as sTREM2 (Schlepckow et al., 2017). The shedding of TREM2 is influenced by various factors. The TREM2 H157Y variant (Schlepckow et al., 2017; Qiao et al., 2023), oligomeric Aβ and the membrane-spanning 4-domain subfamily A (MS4A) gene (Deming et al., 2019) can all increase TREM2 shedding.

A sharp rise in sTREM2 levels disrupts the blood–brain barrier, leading to leakage into the CSF and blood (Raha-Chowdhury et al., 2019), explaining the increased sTREM2 levels in AD and MCI patients. Furthermore, highly sTREM2 expression negatively affects the anti-inflammatory activity of the TREM2 receptor and contributes to disease progression (Dong et al., 2022). Additionally, sTREM2 expression is tightly linked with AD pathology. Significantly elevated sTREM2 levels were observed during Aβ accumulation in the AD mouse model, where it interacts with neurons and plaques (Song et al., 2018). It is reported that sTREM2 levels in CSF are directly correlated with microglial markers (Gispert et al., 2016) and proinflammatory protein levels in the early stages of AD (Rauchmann et al., 2020). Thus, sTREM2 presence is seen as a result of microglial activation and is intimately linked with early-stage neuroinflammation in AD (Nordengen et al., 2019). sTREM2 expression also reflects disease status. As the disease progresses, TREM2 expression in microglial cells varies according to the degree of cell activation, inflammation, and tissue loss.

### 4.3 Association of sTREM2 with recognized AD biomarkers

Numerous studies have established that sTREM2 correlates with key neurodegenerative biomarkers, predominantly Aβ and tau. CSF sTREM2 levels are significantly elevated in AD patients and are strongly correlated with phosphorylated tau (p-tau). Additionally, the correlation between CSF sTREM2 levels and p-tau is a reliable indicator of cognitive decline in older individuals (Ewers et al., 2019). Research indicates that microglial activation accelerates tau protein deposition (Pascoal et al., 2021), and an increase of p-tau181 level is associated with a more rapid increase in CSF sTREM2 (Lan et al., 2024). Moreover, CSF sTREM2 levels fluctuate as AD progresses; Aβ pathology leads to decreased sTREM2 levels, while increases in sTREM2 are associated with Tau deposition (Ma et al., 2020). A recent study also confirmed that plasma Aβ concentrations positively correlate with plasma sTREM2 levels in patients with cognitive impairment (Zhao et al., 2022).

### 4.4 Clinical implications

This is the first meta-analysis confirming that plasma sTREM2 levels are significantly higher in AD patients. The widespread use of CSF biomarkers in clinical practice faces challenges due to lumbar puncture limitations. Recently, clinicians and scientists have increasingly focused on easily accessible and minimally invasive blood biomarkers. It has great potential in the differential diagnosis and tracking of the progression of AD. The development and application of blood biomarkers could transform AD diagnosis and prognosis assessment (Hansson et al., 2022). Published studies on plasma sTREM2 have produced mixed results, but a recent study (Zhao et al., 2022) showed that plasma sTREM2 could serve as a peripheral biomarker to identify cognitive decline in the early stages of neurodegenerative diseases. According to Ohara et al. (2019), a 10-year follow-up indicated a higher risk of dementia in elderly individuals with elevated baseline blood sTREM2 levels. Another study (Min et al., 2021) reported a sensitivity of 81.8% in using plasma sTREM2 to distinguish AD patients from healthy controls. Therefore, more prospective studies are needed to assess the predictive value of plasma sTREM2 for AD progression.

### 4.5 Future perspectives

This study suggests a potential relationship between sTREM2 levels and AD, indicating that sTREM2 could be a biomarker for monitoring disease progression. However, several questions remain unanswered. First, sTREM2 levels are affected by factors such as Braak staging, neuropsychological test scores, and genetic polymorphisms. Future research should stratify AD samples to further explore this association. Second, the study offers insights into the role of gender as a sTREM2 biomarker, but detailed research on the gender-sTREM2-cognitive impairment link is lacking. Future studies should adopt more systematic and comprehensive approaches to examine gender’s influence on this relationship. Finally, continued research on plasma sTREM2 will clarify cutoff values and standards, aiding its use in diagnosing and monitoring cognitive impairment. For consistent and comparable results, standardized experimental methods and uniform laboratory kit and measurement requirements are essential.

### 4.6 Limitations

This study has several limitations. The included studies in the analysis were cross-sectional and case–control, preventing a causal link between sTREM2 levels and AD or MCI. Only two studies focused on plasma sTREM2 levels in MCI patients. The studies showed high heterogeneity, besides the factors listed in our subgroup analysis, other variables like chronic diseases and genetic factors might have affected our findings. Additionally, the existing studies were primarily conducted in European countries and the United States. Therefore, geographic and ethnic influences on our results cannot be dismissed. More research on diverse populations is essential to confirm our findings.

## 5 Conclusion

This study found increased CSF and plasma sTREM2 levels in AD patients, while only CSF sTREM2 levels increased in those with MCI. Currently, no blood test is validated for monitoring brain immune cell activation AD. However, using plasma sTREM2 levels to stratify patient pathology shows tremendous potential. Despite these limitations, our results provide valuable insights into the role of sTREM2 levels in AD progression. Crucially, further validation and detailed functional studies are needed to determine the effects of sTREM2 on AD progression.

## Data availability statement

The original contributions presented in the study are included in the article/Supplementary material, further inquiries can be directed to the corresponding author.

## Author contributions

RW: Data curation, Investigation, Writing – original draft. YZ: Data curation, Investigation, Writing – review & editing. WZ: Methodology, Software, Visualization, Writing – original draft. QY: Methodology, Software, Visualization, Writing – original draft. JP: Conceptualization, Writing – review & editing.
