# Supplementary material for: Association of soluble TREM2 with Alzheimer’s disease and mild cognitive impairment: a systematic review and meta-analysis
Source: Front Aging Neurosci. 2024 May 22;16:1407980. doi: 10.3389/fnagi.2024.1407980 (PMC11150578; doi:10.3389/fnagi.2024.1407980)
Supplement: Supplementary file 1 [file Data_Sheet_1.docx]

Supplementary Material

# Supplementary Tables

**Supplementary Table S1. Search strategy**

| Datebase | Number | Search terms | Items |
| --- | --- | --- | --- |
| Pubmed | #1 | soluble TREM2[Title/Abstract] OR sTREM2[Title/Abstract] OR soluble trigger receptor expressed on myeloid cells 2[Title/Abstract] | 248 |
|  | #2 | Alzheimer’s disease[Title/Abstract] OR Alzheimer[Title/Abstract] OR Senile Dementia[Title/Abstract] OR Dementia[Title/Abstract] OR Alzheimer*[Title/Abstract] OR Cognition disorders[Title/Abstract] OR AD[Title/Abstract] OR Mild Cognitive Impairment[Title/Abstract] OR MCI[Title/Abstract] | 395603 |
|  | #3 | #1 AND #2 | 187 |
| Embase | #1 | 'soluble TREM2':ab,ti OR 'sTREM2':ab,ti OR 'soluble trigger receptor expressed on myeloid cells 2 ':ab,ti | 374 |
|  | #2 | 'Alzheimer Disease':ab,ti OR 'Alzheimer':ab,ti OR 'Senile Dementia ':ab,ti OR 'Cognition disorders ':ab,ti OR 'AD':ab,ti OR 'Mild Cognitive Impairment':ab,ti OR 'MCI':ab,ti | 425622 |
|  | #3 | #1 AND #2 | 268 |
| Cochrane library | #1 | ("soluble TREM2"):ti,ab,kw OR ("sTREM2"):ti,ab,kw OR ("soluble trigger receptor expressed on myeloid cells 2"):ti,ab,kw | 14 |
|  | #2 | ("Alzheimer’s Disease"):ti,ab,kw OR ("Alzheimer"):ti,ab,kw OR ("Senile Dementia"):ti,ab,kw OR ("Cognition disorders"):ti,ab,kw OR ("AD"):ti,ab,kw OR ("Mild Cognitive Impairment"):ti,ab,kw OR ("MCI"):ti,ab,kw | 39440 |
|  | #3 | #1 AND #2 | 9 |
| Web of science | #1 | TS=(‘Alzheimer’s Disease’ OR ‘Alzheimer’ OR ‘Senile Dementia’ OR ‘Dementia’ OR ‘AD’ OR ‘Alzheimer*’ OR ‘Cognition disorders’ OR ‘AD’ OR ‘Mild Cognitive Impairment’ OR ‘MCI’) | 699250 |
|  | #2 | TS=(‘soluble TREM2’ OR ‘sTREM2’ OR ‘soluble trigger receptor expressed on myeloid cells 2’) | 524 |
|  | #3 | #1 AND #2 | 296 |

Supplementary Table S2. Characteristics of the included studies for the meta-analysis

| Study | Year | Country | Diagnostic  criteria | Mean age  (years) | Population | n | Women/men | sTREM2  levels (ng/ml) | Assay  method | Sample  type | Quality  Score |
| --- | --- | --- | --- | --- | --- | --- | --- | --- | --- | --- | --- |
| Finze | 2023 | Germany | NIA-AA | 70.7 (7.4) | AD | 18 | 9/9 | 0.955 (0.088) | ELISA | CSF | 8 |
|  |  |  |  | 69.5 (8.9) | HC | 6 | 3/3 | 1.009 (0.094) |  |  |  |
| Giannisis | 2023 | USA | NIA-AA | 68.6 (8.9) | AD | 24 | 13/11 | 0.352 (0.127) | MSD | CSF | 7 |
|  |  |  |  | 70.1 (6.7) | MCI | 48 | 25/23 | 0.325 (0.105) |  |  |  |
|  |  |  |  | 69.2 (7.2) | HC | 53 | 31/22 | 0.340 (0.124) |  |  |  |
| La Rosa | 2023 | Italy | NINCDS-ADRDA | 77 (5.9) | AD | 15 | 9/6 | 5.189 (0.768) | ELISA | Plasma | 8 |
|  |  |  |  | 71 (6.2) | HC | 12 | 4/8 | 3.681 (0.463） |  |  |  |
| Španić | 2023 | Croatia | NINCDS-ADRDA | 70.58 (8.67) | AD | 155 | NR | 33.854 (19.631) | ELISA | CSF | 8 |
|  |  |  |  | 66.42 (8.83) | MCI | 90 | NR | 26.940 (17.043) |  |  |  |
|  |  |  |  | 58.80 (10.68) | HC | 50 | NR | 18.527 (13.566) |  |  |  |
| Wang | 2023 | ADNI | NINCDS-ADRDA | 74.84 (8.24) | AD | 166 | 67/99 | 4.286 (2.064) | ELISA | CSF | 7 |
|  |  |  |  | 72.81 (6.95) | HC | 676 | 282/394 | 4.027 (1.889) |  |  |  |
| Hok-A-Hin | 2023 | Amsterdam | NIA-AA | 68 (2.0) | AD | 38 | 3/35 | 2.89 (0.63) | ELISA | CSF | 7 |
|  |  |  |  | 67 (1.8) | MCI | 92 | 43/49 | 4.44 (0.80) |  |  |  |
|  |  |  |  | 63 (3.0) | HC | 67 | 16/51 | 3.21 (1.18) |  |  |  |
| Paolini | 2023 | Italy | NIA-AA | 72  (5) | MCI | 25 | 14/11 | 42 (13) | Lumipulse | CSF | 8 |
|  |  |  |  | 64 (14) | HC | 44 | 23/21 | 36 (13) |  |  |  |
| Chen | 2022 | ADNI | NINCDS-ADRDA | 74.2 (8.4) | AD | 85 | 42/43 | 3.465 (2.307) | ELISA | CSF | 7 |
|  |  |  |  | 72.7 (7.1) | MCI | 195 | 81/114 | 3.765 (2.726) |  |  |  |
|  |  |  |  | 73.9 (5.3) | HC | 96 | 38/58 | 3.992 (2.798) |  |  |  |
| Shi | 2022 | ADNI | NIA-AA | 73.36 (7.44) | AD | 85 | 40/45 | 4.864 (2.842) | MSD | CSF | 7 |
|  |  |  |  | 75.90 (5.01) | HC | 46 | 21/25 | 4.495 (2.178) |  |  |  |
| Winfree | 2022 | USA | NIA-AA | 72 (6.18) | MCI | 72 | 26/46 | 3.817 (1.759) | MSD | CSF | 8 |
|  |  |  |  | 72 (6.50) | HC | 83 | 25/58 | 3.530 (1.867) |  |  |  |
| Zhao | 2022 | ADNI | NINCDS-ADRDA | 74.2 (8.7) | AD | 111 | 44/67 | 3.963 (2.082) | MSD | CSF | 8 |
|  |  |  |  | 73.1 (7.4) | MCI | 347 | 149/198 | 3.788 (1.858) |  |  |  |
|  |  |  |  | 73.5 (6.4) | HC | 146 | 74/72 | 3.665 (1.791) |  |  |  |
|  | 2022 | China | NIA-AA | 70.9 (6.0) | AD | 96 | 51/45 | 0.535 (0.188) | ELISA | Plasma | 8 |
|  |  |  |  | 69.6 (6.7) | MCI | 96 | 50/46 | 0.463 (0.162) |  |  |  |
|  |  |  |  | 69.1 (4.3) | HC | 104 | 53/51 | 0.430 (0.218) |  |  |  |
| Li | 2022 | ADNI | NINCDS-ADRDA | 74.6 (8.33) | AD | 198 | 83/115 | 3.84 (2.27) | Elecsys | CSF | 7 |
|  |  |  |  | 72.3 (7.53) | MCI | 527 | 214/313 | 3.69 (3.22) |  |  |  |
|  |  |  |  | 73.7 (5.93) | HC | 310 | 156/154 | 3.69 (1.91) |  |  |  |
| Diaz | 2021 | Spain | NIA-AA | 67 (10) | AD | 35 | 22/13 | 2.896 (1.603) | ELISA | CSF | 6 |
|  |  |  |  | 64 (15) | HC | 48 | 26/22 | 1.898 (0.842) |  |  |  |
| Hu | 2021 | ADNI | NIA-AA | 75.1 (7.8) | AD | 97 | 41/56 | 4.347 (1.975) | MSD | CSF | 6 |
|  |  |  |  | 75.2 (7.6) | MCI | 174 | 62/112 | 4.529 (2.539) |  |  |  |
|  |  |  |  | 75.8 (5.3) | HC | 111 | 54/57 | 4.692 (2.274) |  |  |  |
| Ma | 2021 | ADNI | NIA-AA | 73.78 (5.91) | MCI | 622 | 256/366 | 4.449 (2.285) | MSD | CSF | 7 |
|  |  |  |  | 73.78 (5.91) | HC | 370 | 194/176 | 4.105 (2.123) |  |  |  |
| Schulz | 2021 | USA | NINCDS-ADRDA | 74.27 (4.64) | AD | 11 | 5/6 | 7.750 (3.815) | ELISA | CSF | 8 |
|  |  |  |  | 68.75 (6.38) | HC | 20 | 6/14 | 3.218 (1.463) |  |  |  |
|  | 2021 | USA | NINCDS-ADRDA | 74.27 (4.64) | AD | 11 | 5/6 | 14.108 (6.206) | ELISA | Plasma | 8 |
|  |  |  |  | 68.75 (6.38) | HC | 20 | 6/14 | 3.607 (1.582) |  |  |  |
| Van Hulle | 2021 | UAS | NIA-AA | 72.6 (8.5) | AD | 50 | 18/32 | 9.87 (3.46) | Elecsys | CSF | 6 |
|  |  |  |  | 62.4 (8.4) | HC | 606 | 407/199 | 8.01 (2.46) |  |  |  |
| Banerjee | 2020 | UK | NIA-AA | 62.5 (4.1) | AD | 20 | 11/9 | 6.58 (1.84) | ELISA | CSF | 7 |
|  |  |  |  | 62.2 (5.4) | HC | 10 | 5/5 | 7.96 (2.71) |  |  |  |
| Edwin | 2020 | Norway | NIA-AA | 69.8 (6.5) | AD | 231 | 133/98 | 9.4 (4.6) | ELISA | CSF | 7 |
|  |  |  |  | 71.2 (5.5) | HC | 42 | 25/17 | 8.0 (2.7) |  |  |  |
| Ferri | 2020 | Italy | IWG2 | 78.1 (4.6) | AD | 76 | 53/23 | 39.1 (15.0) | ELISA | Plasma | 7 |
|  |  |  |  | 78.7 (5.6) | HC | 33 | 20/13 | 44.1 (15.1) |  |  |  |
| Franzmeier | 2020 | ADNI | NIA-AA | 74.17 (8.37) | AD | 73 | 35/38 | 4.371 (2.194) | ELISA | CSF | 7 |
|  |  |  |  | 71.82 (7.45) | MCI | 414 | 170/244 | 4.095 (2.105) |  |  |  |
|  |  |  |  | 74.25 (6.08) | HC | 221 | 106/115 | 4.259 (2.184) |  |  |  |
| Knapskog | 2020 | Norway | NIA-AA | 70.1 (6.8) | AD | 237 | 135/102 | 9.5 (4.8) | ELISA | CSF | 8 |
|  |  |  |  | 71.0 (5.4) | MCI | 62 | 36/26 | 9.9 (4.5) |  |  |  |
|  |  |  |  | 72.3 (6.0) | HC | 113 | 54/59 | 8.8 (3.6) |  |  |  |
| Deming | 2019 | ADNI | NINCDS-ADRDA | 74.39 (8.56) | AD | 172 | 74/98 | 4.019 (1.946) | MSD | CSF | 7 |
|  |  |  |  | 71.23 (7.39) | MCI | 183 | 77/106 | 3.742 (2.066) |  |  |  |
|  |  |  |  | 74.47 (5.85) | HC | 169 | 80/89 | 3.988 (1.923) |  |  |  |
| Ewers | 2019 | ADNI | NIA-AA | 73.6 (8.51) | AD | 66 | 32/34 | 4.608 (2.201) | ELISA | CSF | 7 |
|  |  |  |  | 72.9 (7.11) | MCI | 184 | 77/107 | 4.452 (2.518) |  |  |  |
|  |  |  |  | 72.8 (5.36) | HC | 100 | 45/55 | 3.762 (1.841) |  |  |  |
| Morenas | 2019 | Spain | NIA-AA | 74.6 (5.6) | AD | 50 | 31/19 | 4.3 (2.2) | ELISA | CSF | 8 |
|  |  |  |  | 67.4 (5.1) | HC | 44 | 25/19 | 4.2 (2.3) |  |  |  |
| Nordengen | 2019 | Norway | NIA-AA | 67.6 (5.2) | AD | 27 | 13/14 | 4.8 (1.7) | ELISA | CSF | 8 |
|  |  |  |  | 66.6 (7.4) | MCI | 40 | 23/17 | 4.0 (1.8) |  |  |  |
|  |  |  |  | 61.1 (9.2) | HC | 36 | 19/17 | 3.1 (0.9) |  |  |  |
| Brosseron | 2018 | Germany | NIA-AA | 74 (8) | AD | 116 | 45/71 | 4.315 (2.235) | MSD | CSF | 7 |
|  |  |  |  | 71 (8) | MCI | 130 | 65/65 | 4.07 (2.54) |  |  |  |
|  |  |  |  | 67 (11) | HC | 85 | 65/20 | 2.994 (2.269) |  |  |  |
| Bekris | 2018 | USA | NIA-AA | 66 (7) | AD | 83 | 42/41 | 3.0 (0.185) | Luminex | CSF | 7 |
|  |  |  |  | 67 (9) | MCI | 38 | 17/21 | 3.1 (0.283) |  |  |  |
|  |  |  |  | 62 (12) | HC | 37 | 16/21 | 3.0 (0.215) |  |  |  |
| Bekris | 2018 | USA | NIA-AA | 66 (7) | AD | 83 | 40/43 | 2.9 (0.255) | Luminex | Plasma | 7 |
|  |  |  |  | 67 (9) | MCI | 35 | 15/20 | 3.05 (0.345) |  |  |  |
|  |  |  |  | 62 (13) | HC | 104 | 50/54 | 2.9 (0.255) |  |  |  |
| Gispert | 2017 | Spain | NIA-AA | 67.1 (11.3) | AD | 15 | 11/4 | 0.5866 (0.3589) | ELISA | CSF | 8 |
|  |  |  |  | 61.9 (8.8) | HC | 49 | 32/17 | 0.4235 (0.2176) |  |  |  |
| Gispert | 2016 | Spain | NIA-AA | 66.78 (9.75) | AD | 23 | 16/7 | 3.34 (1.53) | ELISA | CSF | 8 |
|  |  |  |  | 70.30 (7.35) | MCI | 27 | 15/12 | 4.16 (1.97) |  |  |  |
|  |  |  |  | 60.98 (6.83) | HC | 45 | 28/17 | 2.40 (1.14) |  |  |  |
| Henjum | 2016 | Sweden | NINCDS-ADRDA | 79 (6.3) | AD | 25 | 18/7 | 3.80 (2.20) | ELISA | CSF | 7 |
|  |  |  |  | 62 (9.3) | HC | 25 | 17/8 | 3.20 (1.63) |  |  |  |
|  | 2016 | Norway | NIA-AA | 68 (4.8) | AD | 29 | 13/16 | 4.80 (2.67) | ELISA | CSF | 7 |
|  |  |  |  | 67 (5.0) | MCI | 21 | 12/9 | 4.10 (2.59) |  |  |  |
|  |  |  |  | 66 (9.0) | HC | 50 | 25/25 | 4.40 (2.00) |  |  |  |
| Heslegrave | 2016 | UK | IWG2 | 70.51 (7.5) | AD | 37 | 19/18 | 0.231 (0.098) | UPLC-MS | CSF | 8 |
|  |  |  |  | 69.2 (8.0) | HC | 22 | 10/12 | 0.196 (0.081) |  |  |  |
| Heslegrave | 2016 | Sweden | IWG2 | 64.3 (6.8) | AD | 24 | 13/11 | 0.231 (0.097) | UPLC-MS | CSF | 8 |
|  |  |  |  | 55.6 (9.7) | HC | 16 | 9/7 | 0.173 (0.065) |  |  |  |
| Piccio | 2016 | Europe | NINCDS-ADRDA | 76.6 (5.2) | AD | 73 | 36/37 | 1.028 (0.582) | ELISA | CSF | 7 |
|  |  |  |  | 70.2 (8.5) | HC | 107 | 57/50 | 0.832 (0.508) |  |  |  |
|  | 2016 | Europe | NINCDS-ADRDA | 76.6 (5.2) | AD | 73 | 36/37 | 1.019 (0.589) | ELISA | Plasma | 7 |
|  |  |  |  | 70.2 (8.5) | HC | 107 | 57/50 | 0.976 (0.603) |  |  |  |
| Suárez-Calvet | 2016 | Europe | NIA-AA | 73.8 (10) | AD | 200 | 62/138 | 5.33 (3.7) | ELISA | CSF | 8 |
|  |  |  |  | 74.3 (9) | MCI | 111 | 60/51 | 5.98 (3.2) |  |  |  |
|  |  |  |  | 62.4 (11) | HC | 150 | 59/91 | 3.07 (1.4) |  |  |  |
| Hu | 2014 | China | NINCDS-ADRDA | 79.59 (7.46) | AD | 116 | 59/57 | 20.97 (7.25) | ELISA | Plasma | 8 |
|  |  |  |  | 79.28 (7.84) | HC | 116 | 57/59 | 19.63 (6.84) |  |  |  |
| Kleinberger | 2014 | Germany | NINCDS-ADRDA | 70.4 (8.9) | AD | 56 | 38/18 | 1.950 (1.099) | ELISA | CSF | 7 |
|  |  |  |  | 60.7 (9.5) | HC | 88 | 55/33 | 2.217 (1.064) |  |  |  |
|  | 2014 | Germany | NINCDS-ADRDA | 70.7 (9.0) | AD | 51 | 36/15 | 7.276 (1.893) | ELISA | Plasma | 7 |
|  |  |  |  | 60.7 (9.5) | HC | 88 | 55/33 | 2.217 (1.064) |  |  |  |

ADNI, Alzheimer’s Disease Neuroimaging Initiative; NIA-AA, National Institute on Aging-Alzheimer’s Association; NINCDS-ADRDA, National Institute of Neurological and Communicative Disorders and Stroke-Alzheimer Disease and Related Disorders Association; IWG2, the revised proposed International Working Group; AD, Alzheimer’s disease; MCI, mild cognitive impairment; HC, healthy control; NR, not reported; ELISA, enzyme-linked immunosorbent assay; MSD, mesoscale discovery electrochemiluminescence platform-based assay; Elecsys, electrochemiluminescence immunoassay; UPLC-MS, ultra-performance liquid chromatography-tandem mass spectrometer; CSF, cerebrospinal fluid.

**Supplementary Table S3. Meta-regression of CSF or plasma sTREM2 levels in patients with AD and MCI**

| Comparison | Sample type | Mean age | | Proportion of women | | Diagnostic criteria | | Assay method | |
| --- | --- | --- | --- | --- | --- | --- | --- | --- | --- |
|  |  | *P*＞\|t\| | Coef. (95%CI) | *P*＞\|t\| | Coef. (95%CI) | *P*＞\|t\| | Coef.  (95%CI) | *P*＞\|t\| | Coef. (95%CI) |
| AD vs. HC | CSF | 0.888 | 0.03  (-0.38, 0.44) | 0.192 | -0.24  (-0.60, 0.13) | 0.920 | -0.02  (-0.41, 0.37) | 0.800 | -0.04  (-0.30, 0.39) |
| MCI vs. HC | CSF | 0.508 | 0.19  (-0.41, 0.79) | 0.766 | -0.07  (-0.45, 0.60) | 0.231 | -0.35  ( -0.95, 0.25) | 0.433 | -0.18  (-0.66, 0.30) |
| AD vs. MCI | CSF | 0.187 | 0.47  (-0.27, 1.22) | 0.163 | 0.55  (-0.26, 1.35) | 0.857 | -0.07  (-0.86, 0.73) | 0.364 | -0.28  (-0.92, 0.37) |
| AD vs. HC | Plasma | 0.170 | -2.58  (-6.87, 1.71) | 0.104 | 1.67  (-0.54, 3.88) | 0.622 | -0.58  (-3.58, 2.42) | - | - |

**Supplementary Table S4. Egger's test of CSF or plasma sTREM2 levels in patients with AD and MCI**

| Comparison | Sample type | Std_Eff | Coef. | Std. Err. | t | *P*>\|t\| | 95% CI |
| --- | --- | --- | --- | --- | --- | --- | --- |
| AD vs. HC | CSF | Slope | 0.07 | 0.16 | 0.45 | 0.654 | -0.26, 0.41 |
|  |  | bias | 1.09 | 1.00 | 1.08 | 0.290 | -0.98, 3.12 |
| MCI vs. HC | CSF | Slope | -0.20 | 0.16 | -1.06 | 0.305 | -0.59, 0.19 |
|  |  | bias | 3.28 | 1.50 | 2.19 | 0.042 | 0.13, 6.44 |
| AD vs. MCI | CSF | Slope | -0.28 | 0.26 | -1.07 | 0.300 | -0.84, 0.28 |
|  |  | bias | 2.32 | 1.89 | 1.23 | 0.239 | -1.71, 6.35 |
| AD vs. HC | Plasma | Slope | -0.74 | 0.43 | -1.71 | 0.138 | -1.79, 0.30 |
|  |  | bias | 5.32 | 2.44 | 2.18 | 0.072 | -0.65, 11.30 |

# Supplementary Figures

**
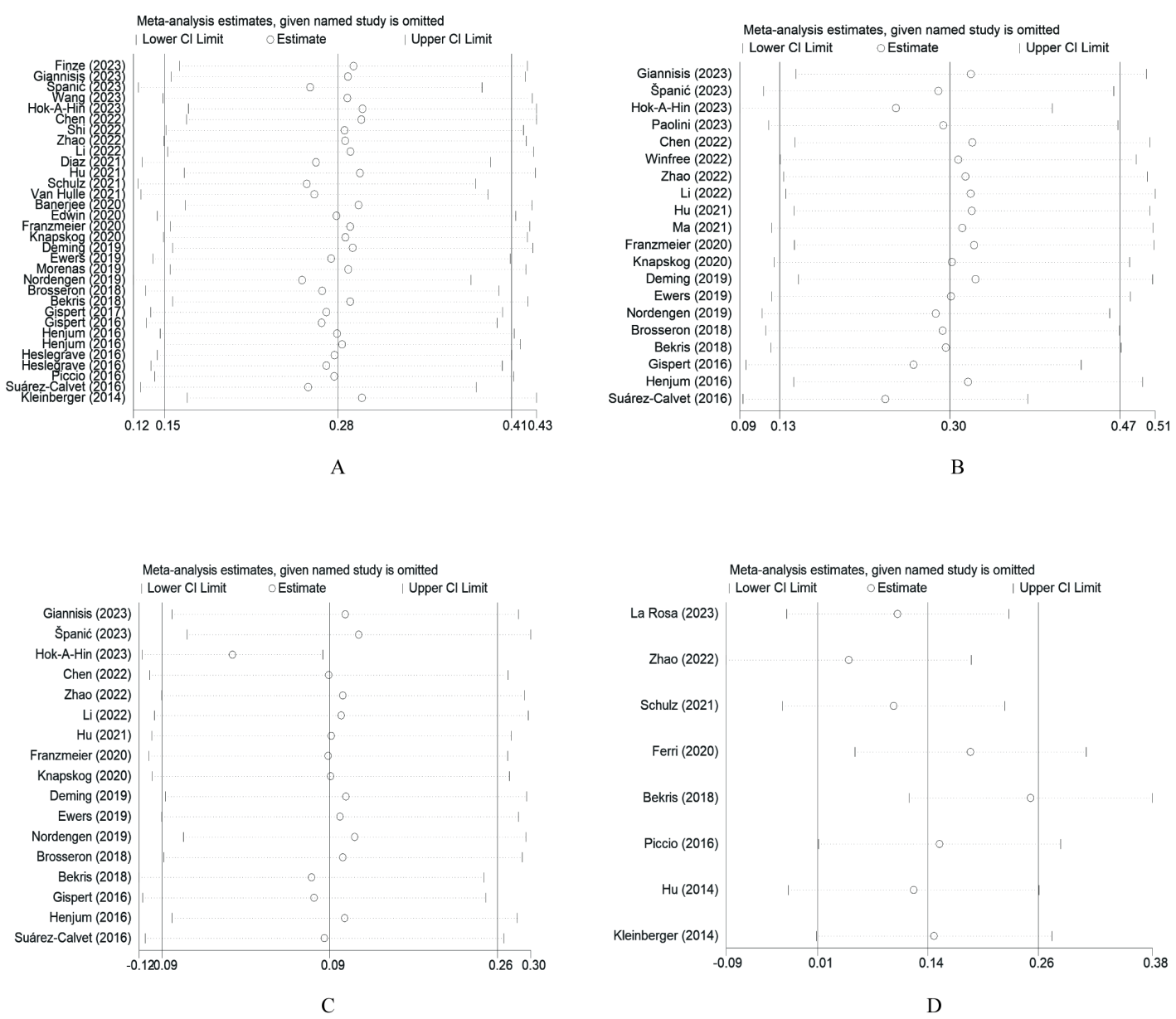
**

**Supplementary Figure S1. The results of the sensitivity analysis of the sTREM2 level with AD and MCI risk. (A) AD vs. HC in CSF; (B) MCI vs. HC in CSF; (C) AD vs. MCI in CSF; (D) AD vs. HC in plasma.**

**
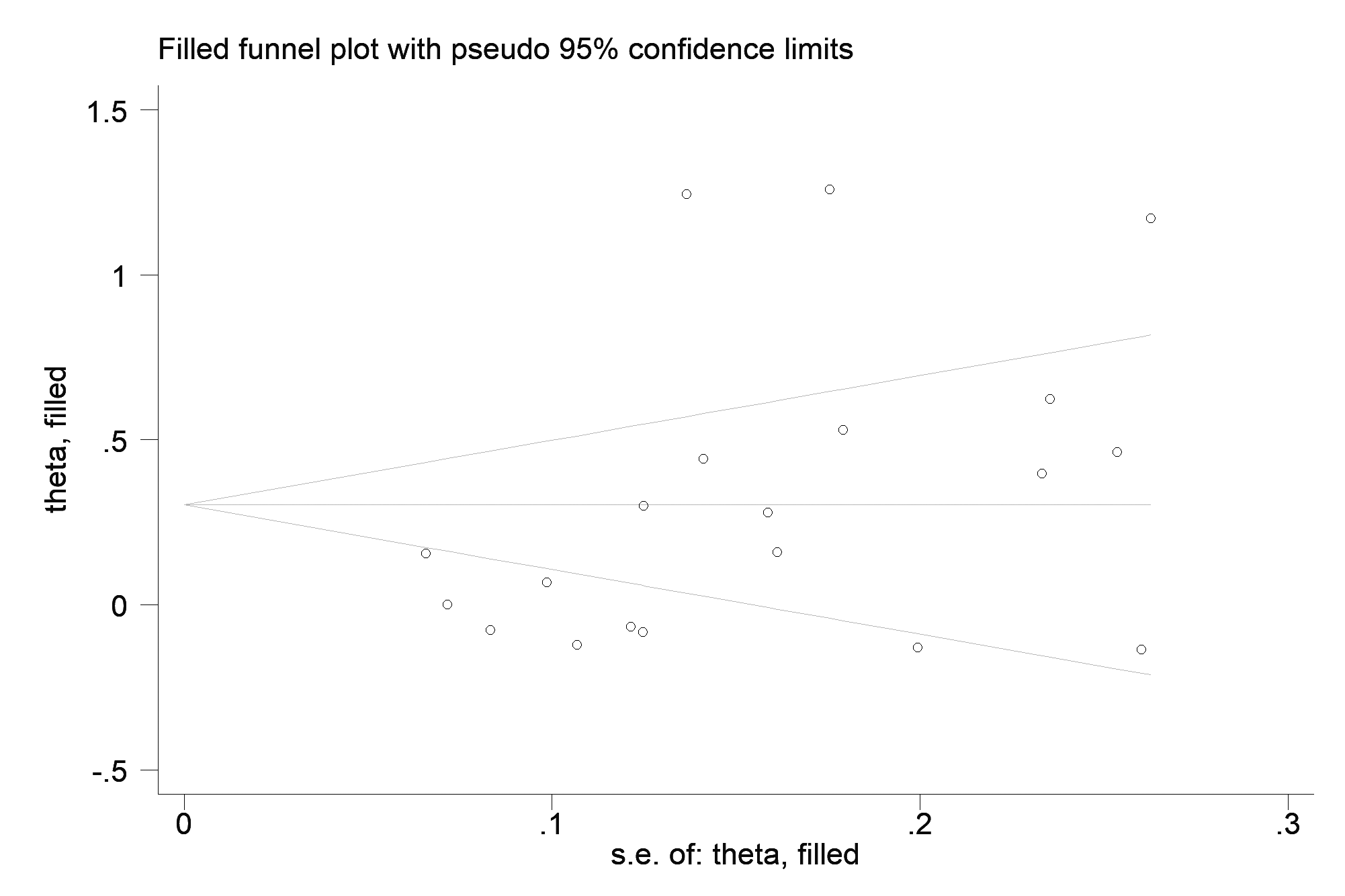
**

**Supplementary Figure S2. Funnel plot detailing publication bias in the studies investigating the CSF sTREM2 levels in the patients with MCI and control group after trimming and filling.**
